# Supplementary material for: Risk of hospitalized and non-hospitalized gastrointestinal bleeding in ALLHAT trial participants receiving diuretic, ACE-inhibitor, or calcium-channel blocker
Source: PLoS One. 2021 Nov 18;16(11):e0260107. doi: 10.1371/journal.pone.0260107 (PMC8601451; doi:10.1371/journal.pone.0260107)
Supplement: S1 Table — (PDF) [file pone.0260107.s001.pdf]

Table S1. ICD-9 and ICD-10 Codes for GI Bleeding and Convert ICD-9-CM Codes to ICD-10-CM*

ICD-9 Codes	Description	ICD-10 Codes	Description
531.0+0/1	Acute gastric ulcer with hemorrhage	K25.0	Acute gastric ulcer with hemorrhage
531.2+0/1	Acute gastric ulcer with hemorrhage and perforation	K25.2	Acute gastric ulcer with both hemorrhage and perforation
531.4+0/1	Chronic or unspecified gastric ulcer with hemorrhage	K25.4	Chronic or unspecified gastric ulcer with hemorrhage
531.6+0/1	Chronic or unspecified gastric ulcer with hemorrhage and perforation	K25.6	Chronic or unspecified gastric ulcer with both hemorrhage and perforation
532.0+0/1	Acute duodenal ulcer with hemorrhage	K26.0	Acute duodenal ulcer with hemorrhage
532.2+0/1	Acute duodenal ulcer with hemorrhage and perforation	K26.2	Acute duodenal ulcer with both hemorrhage and perforation
532.4+0/1	Chronic or unspecified duodenal ulcer with hemorrhage	K26.4	Chronic or unspecified duodenal ulcer with hemorrhage
532.6+0/1	Chronic or unspecified duodenal ulcer with hemorrhage and perforation	K26.6	Chronic or unspecified duodenal ulcer with both hemorrhage and perforation
533.0+0/1	Acute peptic ulcer of unspecified site with hemorrhage	K27.0	Acute peptic ulcer, site unspecified, with hemorrhage
533.2+0/1	Acute peptic ulcer of unspecified site with hemorrhage and perforation	K27.2	Acute peptic ulcer, site unspecified, with both hemorrhage and perforation
533.4+0/1	Chronic or unspecified peptic ulcer of unspecified site with hemorrhage	K27.4	Chronic or unspecified peptic ulcer, site unspecified, with hemorrhage
533.6+0/1	Chronic or unspecified peptic ulcer of unspecified site with hemorrhage and perforation	K27.6	Chronic or unspecified peptic ulcer, site unspecified, with both hemorrhage and perforation
534.0+0/1	Acute gastrojejunal ulcer with hemorrhage	K28.0	Acute gastrojejunal ulcer with hemorrhage
534.2+0/1	Acute gastrojejunal ulcer with hemorrhage and perforation	K28.2	Acute gastrojejunal ulcer with both hemorrhage and perforation
534.4+0/1	Chronic or unspecified gastrojejunal ulcer with hemorrhage	K28.4	Chronic or unspecified gastrojejunal ulcer with hemorrhage
534.6+0/1	Chronic or unspecified gastrojejunal ulcer with hemorrhage and perforation	K28.6	Chronic or unspecified gastrojejunal ulcer with both hemorrhage and perforation
535.01	Acute gastritis, with hemorrhage	K29.01	Acute gastritis with bleeding
535.11	Atrophic gastritis, with hemorrhage	K29.41, K29.51	Chronic atrophic gastritis with bleeding, Unspecified chronic gastritis with bleeding
535.21	Gastric mucosal hypertrophy, with hemorrhage	K29.61	Other gastritis with bleeding
535.31	Alcoholic gastritis, with hemorrhage	K29.21	Alcoholic gastritis with bleeding
535.41	Other specified gastritis, with hemorrhage	K29.61	Other gastritis with bleeding
535.51	Unspecified gastritis and gastroduodenitis, with hemorrhage	K29.71	Gastritis, unspecified, with bleeding, or
	Same as above (ICD-9 535.51)	K29.91	Gastroduodenitis, unspecified, with bleeding
535.61	Duodenitis, with hemorrhage	K29.81	Duodenitis with bleeding
535.71	Eosinophilic gastritis, with hemorrhage	K52.81	Eosinophilic gastritis or gastroenteritis
578.0	Gastrointestinal haemorrhage, Hematemesis	K92.0	Hematemesis
578.1	Gastrointestinal haemorrhage, Blood in stool	K92.1	Melena (Blood in stool)
578.9	Hemorrhage of gastrointestinal tract, unspecified	K92.2	Gastrointestinal hemorrhage, unspecified

*Convert source online: https://www.icd10data.com/Convert.
