# Supplementary material for: Risk of hospitalized and non-hospitalized gastrointestinal bleeding in ALLHAT trial participants receiving diuretic, ACE-inhibitor, or calcium-channel blocker
Source: PLoS One. 2021 Nov 18;16(11):e0260107. doi: 10.1371/journal.pone.0260107 (PMC8601451; doi:10.1371/journal.pone.0260107)
Supplement: S1 Text — (PDF) [file pone.0260107.s002.pdf]

S1 Text. Supplement File on Statistical Programs and Models:

Programs on identifying gastrointestinal (GI) bleeding (GI) bleeding:

```
stset GIB3year if GIBleed3InpPre < ., failure(GIBleed3InpPre)
*
foreach SubgVar of varlist GIBleed3InpPre Black Hisp Male AspirinBL Age65 Age6569 Age70
Smoker {
  foreach SubgCode in 0 1 {
    disp as input "{hline}"
    disp "{right:Population: `:label(`SubgVar') `SubgCode''}"
    disp "{right:Chlorthalidone vs. Amlodipine}"
    disp "{hline}"
          tab GIBleed3InpPre RZGRP if `SubgVar' == `SubgCode' & ChlorAmlod<. ///
                  & GIB3year>0 & GIB3year<.
    stcox ChlorAmlod if `SubgVar' == `SubgCode'
*
*--- do my version rounded to 5.2f
    disp as input  _newline "{hline}" _newline ///
     "{center:Numbers repeat below rounded to 2 decimal places} "
    pg_coxout
*
    disp as input "{hline}"
    disp "{right:Population: `:label(`SubgVar') `SubgCode''}"
    disp "{right:Lisinopril vs. Amlodipine}"
    disp "{hline}"
          tab GIBleed3InpPre RZGRP if `SubgVar' == `SubgCode' & LisinAmlod<. ///
                  & GIB3year>0 & GIB3year<.
    stcox LisinAmlod if `SubgVar' == `SubgCode'
*
    disp as input  _newline "{hline}" _newline ///
     "{center:Numbers repeat below rounded to 2 decimal places} "
    pg_coxout
*
    disp as input "{hline}"
    disp "{right:Population: `:label(`SubgVar') `SubgCode''}"
    disp "{right:Lisinopril vs. Chlorthalidone}"
    disp "{hline}"
          tab GIBleed3InpPre RZGRP if `SubgVar' == `SubgCode' & LisinChlor<. ///
                  & GIB3year>0 & GIB3year<.
    stcox LisinChlor if `SubgVar' == `SubgCode'
*
    disp as input  _newline "{hline}" _newline ///
     "{center:Numbers repeat below rounded to 2 decimal places} "
    pg_coxout
  }
  disp as input "{hline}"
  disp "{right:Chlorthalidone vs. Amlodipine}"
  disp "{hline}"
  stcox ChlorAmlod `SubgVar'
```

```
*
*--- do my version rounded to 5.2f
   disp as input  _newline "{hline}" _newline ///
    "{center:Numbers repeat below rounded to 2 decimal places} "
   pg_coxout
*
   generate ChlAmlX`SubgVar' = ChlorAmlod * `SubgVar'
   stcox ChlorAmlod `SubgVar' ChlAmlX``SubgVar''
   drop ChlAmlX``SubgVar''
   disp as input  _newline "{hline}" _newline ///
    "{center:Numbers repeat below rounded to 2 decimal places} "
   pg_coxout
*
   disp as input "{hline}"
   disp "{right:Lisinopril vs. Amlodipine}"
   disp "{hline}"
   stcox LisinAmlod `SubgVar'
*
   disp as input  _newline "{hline}" _newline ///
    "{center:Numbers repeat below rounded to 2 decimal places} "
   pg_coxout
*
   generate LisAmlX`SubgVar' = LisinAmlod * `SubgVar'
   stcox LisinAmlod `SubgVar' LisAmlX`SubgVar'
   drop LisAmlX`SubgVar'
*
   disp as input  _newline "{hline}" _newline ///
    "{center:Numbers repeat below rounded to 2 decimal places} "
   pg_coxout
*
   disp as input "{hline}"
   disp "{right:Lisinopril vs. Chlorthalidone}"
   disp "{hline}"
   stcox LisinChlor `SubgVar'
*
   disp as input  _newline "{hline}" _newline ///
    "{center:Numbers repeat below rounded to 2 decimal places} "
   pg_coxout
*
   generate LisChlX`SubgVar' = LisinChlor * `SubgVar'
   stcox LisinChlor `SubgVar' LisChlX`SubgVar'
   drop LisChlX`SubgVar'
*
   disp as input  _newline "{hline}" _newline ///
    "{center:Numbers repeat below rounded to 2 decimal places} "
   pg_coxout

}
```

<u>Table 3 Cox Sample:</u>

Total - Hospitalized Chlorthalidone vs. Amlodipine

```
------------------------------------------------------------------------------------

  GI Bleed |
Occurred-d |
 ef3 up to |
 31mar2002 |   Antihypertensive
 (Combined |     Treatment Grp
Inpatient) | Chlorthal  Amlodipin |     Total
-----------+----------------------+----------
       Yes |       411        244 |       655
-----------+----------------------+----------
     Total |       411        244 |       655


         failure _d:  GIBleed3InpPre
   analysis time _t:  GIB3year

Iteration 0:   log likelihood =  -3597.356
Iteration 1:   log likelihood = -3597.3172
Iteration 2:   log likelihood = -3597.3172
Refining estimates:
Iteration 0:   log likelihood = -3597.3172

Cox regression -- Breslow method for ties

No. of subjects =          655                  Number of obs    =         655
No. of failures =          655
Time at risk    =  2092.900758
                                                LR chi2(1)       =        0.08
Log likelihood  =   -3597.3172                  Prob > chi2      =      0.7806

------------------------------------------------------------------------------
          _t | Haz. Ratio   Std. Err.      z    P>|z|     [95% Conf. Interval]
-------------+----------------------------------------------------------------
  ChlorAmlod |     .97769   .0791227    -0.28   0.780      .834286    1.145743
```

Lisinopril vs. Amlodipine

```
------------------------------------------------------------------------------------
  GI Bleed |
Occurred-d |
 ef3 up to |
 31mar2002 |   Antihypertensive
 (Combined |     Treatment Grp
Inpatient) | Amlodipin  Lisinopri |     Total
-----------+----------------------+----------
       Yes |       244        259 |       503
```

```
-----------+----------------------+----------
    Total |       244        259 |       503


        failure _d:  GIBleed3InpPre
   analysis time _t:  GIB3year

Iteration 0:   log likelihood = -2630.2506
Iteration 1:   log likelihood = -2630.2139
Iteration 2:   log likelihood = -2630.2139
Refining estimates:
Iteration 0:   log likelihood = -2630.2139

Cox regression -- Breslow method for ties

No. of subjects =          503                   Number of obs    =         503
No. of failures =          503
Time at risk    =  1571.797402
                                                  LR chi2(1)       =        0.07
Log likelihood  =   -2630.2139                    Prob > chi2      =      0.7862

------------------------------------------------------------------------------
         _t | Haz. Ratio   Std. Err.      z    P>|z|     [95% Conf. Interval]
-------------+----------------------------------------------------------------
  LisinAmlod |   .9759683   .0875342    -0.27   0.786     .8186379    1.163535
------------------------------------------------------------------------------


Lisinopril vs. Chlorthalidone
--------------------------------------------------------------------------------

  GI Bleed |
Occurred-d |
 ef3 up to |
 31mar2002 |   Antihypertensive
 (Combined |     Treatment Grp
Inpatient) | Chlorthal  Lisinopri |     Total
-----------+----------------------+----------
       Yes |       411        259 |       670
-----------+----------------------+----------
     Total |       411        259 |       670


        failure _d:  GIBleed3InpPre
   analysis time _t:  GIB3year

Iteration 0:   log likelihood = -3694.5326
Iteration 1:   log likelihood = -3694.5308
Iteration 2:   log likelihood = -3694.5308
Refining estimates:
Iteration 0:   log likelihood = -3694.5308
```

Cox regression -- Breslow method for ties

No. of subjects = 670 Number of obs = 670
No. of failures = 670
Time at risk = 2124.350449
LR chi2(1) = 0.00
Log likelihood = -3694.5308 Prob > chi2 = 0.9516

_t	Haz. Ratio	Std. Err.	z	P>|z|	[95% Conf. Interval]	
LisinChlor	.9951619	.0795323	-0.06	0.952	.8508766	1.163914

Table 4 KM Estimates:

K-M estimates for GI Bleed Occurred-def3 up to 31mar2002 (Combined Inpatient)
Population: Total
Groups: Chlor, Amlod, Lisin
----

failure _d: GIBleed3InpPre
analysis time _t: GIB3year

Time	Beg. Total	Fail	Failure Function	Std. Error	[95% Conf.	Int.]
Chlor						
0	0	0	0.0000	.	.	.
1	7611	49	0.0064	0.0009	0.0048	0.0085
2	7542	70	0.0155	0.0014	0.0130	0.0186
3	7471	71	0.0248	0.0018	0.0216	0.0285
4	7399	71	0.0341	0.0021	0.0302	0.0384
5	4851	82	0.0470	0.0025	0.0424	0.0521
6	2522	47	0.0593	0.0031	0.0536	0.0656
Amlod						
0	0	0	0.0000	.	.	.
1	4506	32	0.0071	0.0012	0.0050	0.0100
2	4462	44	0.0168	0.0019	0.0134	0.0209
3	4426	36	0.0247	0.0023	0.0206	0.0296
4	4375	51	0.0359	0.0028	0.0309	0.0418
5	2863	42	0.0469	0.0032	0.0410	0.0536
6	1547	23	0.0565	0.0038	0.0496	0.0643
Lisin						
0	0	0	0.0000	.	.	.
1	4439	42	0.0094	0.0014	0.0069	0.0127
2	4399	40	0.0183	0.0020	0.0148	0.0227
3	4357	42	0.0277	0.0025	0.0233	0.0329
4	4307	50	0.0388	0.0029	0.0336	0.0449
5	2806	42	0.0500	0.0033	0.0438	0.0569
6	1463	25	0.0612	0.0040	0.0538	0.0695

Note: Failure function is calculated over full data and evaluated at indicated times; it is not calculated from aggregates shown at left.

And a sample of the KM code loop:

```
*
foreach grp in dummy {
   disp as input "{hline}"
   disp "{right:K-M estimates for `:var label GIBleed3InpPre'}"
   disp "{right:Population: Total}"
   disp "{hline}"
   stset GIB3year if GIBleed3InpPre < ., failure(GIBleed3InpPre)
```

```
   disp as input "{hline}"
   disp "{right:Population: Total}"
   disp "{right:Groups: Total}"
   disp "{hline}"
   sts list, failure at(0 1 2 3 4 5 6)
   disp as input "{hline}"
   disp "{right:Population: Total}"
   disp "{right:Groups: Chlor, Amlod, Lisin}"
   disp "{hline}"
   sts list, by(Group) failure at(0 1 2 3 4 5 6)
*
```

<u>Statistical models on the Kaplan-Meier estimates and Cox regressions using STATA software which were used in this specific manuscript:</u>

1). Kaplan Meier estimates on the cumulative incidence of GI bleeding:

```
sts list, by(Group) failure at(0 1 2 3 4 5 6)
```

2). Cox regressions on the hazard ratio of GI bleeding:

```
stset Outcomeyear if Outcome < ., failure(Outcome)

stcox GroupComparison
```
