# Supplementary material for: Risk of hospitalized and non-hospitalized gastrointestinal bleeding in ALLHAT trial participants receiving diuretic, ACE-inhibitor, or calcium-channel blocker
Source: PLoS One. 2021 Nov 18;16(11):e0260107. doi: 10.1371/journal.pone.0260107 (PMC8601451; doi:10.1371/journal.pone.0260107)
Supplement: Attachment — Submitted filename: Response to reviewers (PlosOne).docx [file pone.0260107.s003.docx]

August 3, 2021

James M Wright
Academic Editor
PLOS ONE

Re: Manuscript

PONE-D-21-20128
“Risk of hospitalized and non-hospitalized gastrointestinal bleeding in ALLHAT trial participants receiving diuretic, ACE-inhibitor, or calcium-channel blocker”

Dear Dr. Wright:

Thank you and reviewers for very helpful comments. We have made all the changes as suggested, which are highlighted (in track changes) in the revised manuscript. Below is a list of responses to the reviewer’s comments on a point-by-point basis.

We hope these revisions are satisfactory to you and the reviewers. We look forward to hearing from you soon.

Sincerely,

Xianglin L. Du, M.D., Ph.D.

**Responses to the reviewer’s comments on a point-by-point basis.**

Reviewer #1: This is a useful study that further adds to the analysis of the ALLHAT study, showing that there is no significant difference between the antihypertensive medications used in the 3 arms of the trial regarding the incidence of GI bleeding. Through the use linked ALLHAT participant Medicare claims data taken to the end of the study, the authors were able to conduct a data linkage analysis that supports the conclusion about GI bleeding and the conclusion that age and smoking is associated to increased risk of GI bleeding while other factors are associated with reduced risk of GI bleeding. While conclusions drawn from the ALLHAT trial and accompanying analyses will always be limited by the rapidly updating clinical evidence, this study is important in expanding our understanding of the results of the trial for better completeness and for reviews of the existing evidence.

Major concerns
1. In Data Availability, relevant to the Methods section, the authors state “We plan to share the statistical models and statistical programs that we sued to analyze these data upon request”. The statistical programs and the versions used for the Kaplan-Meier method and the Cox regression models should be provided at the very least in the Statistical analysis section found on page 7.

**Response:** As suggested, we have added our statistical programs for the Kaplan-Meier estimates on the cumulative incidence of GI bleeding and the Cox regressions on the hazard ratio of GI bleeding in the “Data Availability” section.

Minor concerns
1. Authors state that the smoking group are at increased risk of GI bleed, and this is supported in Table 5 but only for hospitalized GI bleeding, it should be specified in the Results page 10 second paragraph “Smokers also had a significantly higher risk of having GI bleeding than those who did not” should be “Smokers also had a significantly higher risk of having hospitalized GI bleeding than those who did not”. Page 2 Results paragraph “Smokers also had a significantly higher risk of having GI bleeding” should be “Smokers also had a significantly higher risk of having hospitalized GI bleeding”.

**Response:** Thank you for helpful suggestions. We have made these two changes as suggested.

2. The generalized conclusion statements about the relevance of this study for its clinical significance should be more specific. Page 5 first paragraph “Hence, the findings of this study should have high public health and clinical significance in terms of overall side effects and routine intake of antihypertensive drugs” should be “Hence, the findings of this study should have high public health and clinical significance with respect to GI bleeding and routine intake of antihypertensive drugs”. Page 12 paragraph one, line 4 “health implications for the safety and side-effects of antihypertensive drugs,” should be “health implications for the safety and side effects of antihypertensive drugs regarding GI bleeding,”

**Response:** Thank you for helpful suggestion. We have made this change as suggested on page 5.

3. Minor grammatical clarification on page 12, paragraph one, line 5 “study’s follow-up time was back to March 2002.” should be “study’s follow-up time was dated back to March 2002.”

**Response:** Thank you for helpful suggestion. We have made this change as suggested on page 12.

Reviewer #2: This is a very clear and simple study with important conclusions. I am not competent to judge the statistical methods used, but the study description, patient characteristics and findings, including limitations are all presented clearly and completely.
It should be published without any revisions.

**Response:** Thank you so much for positive comments!
